# Gut-to-tumor translocation of multidrug-resistant *Klebsiella pneumoniae* shapes the microbiome and chemoresistance in pancreatic cancer

**DOI:** 10.3389/fcimb.2025.1694479

**Published:** 2025-12-02

**Authors:** Lei Zhao, Shifu Peng, Muxi Ge, Boming Xing, Xinyang Zhao, Tongquan Yang, Shenghui Yu, Cheng Zhang, Jinyang Liu, Ziwei Miao, Heyao Ma

**Affiliations:** 1 Department of Hepatobiliary and Pancreatic Surgery, First Hospital of China Medical University, Shenyang, Liaoning, China; 2 Department of Environment and Health, Jiangsu Center for Disease Control and Prevention, Nanjing, China; 3 The First Clinical College of China Medical University, Shenyang, Liaoning, China; 4 Research Institute for Cancer Therapy, First Hospital of China Medical University, Shenyang, Liaoning, China; 5 Department of Developmental Cell Biology, Key Laboratory of Medical Cell Biology, Ministry of Education, China Medical University, Shenyang, China; 6 Department of Pharmacology, School of Pharmacy, China Medical University, Shenyang, China

**Keywords:** antibiotic resistance gene (ARG), gut microbiome, pancreatic cancer, whole-genome sequencing, metagenomic analysis, *Klebsiella pneumoniae*

## Abstract

**Background:**

Despite advances and successes in precision oncology, pancreatic cancer (PC) remains a tumor with extremely low survival rates, and many of these cases experienced postoperative recurrence and metastasis. Alterations in the gut microbiota have been linked to the survival rates of PC patients. Nevertheless, the complexity of gut microbiota composition poses significant challenges in identifying definitive clinical biomarkers for PC.

**Methods:**

Fecal samples were collected from PC patients, half of whom had metastasis, and their matched healthy controls (HCs). A metagenomic analysis was employed to further investigate the functional features of gut microbiota with both PC and metastatic PC. The clinical correlations, microbial metabolic pathways and antibiotic resistome were further assessed. In a follow-up validation, intraoperative tumor tissue and pancreatic fluid were sampled from PC patients and underwent comprehensive microbiological analysis, including bacterial culture, mass spectrometry-based identification, and third-generation whole-genome sequencing of *Klebsiella pneumoniae* isolates.

**Results:**

We observed a significant alteration of the gut microbiota in PC patients, highlighted by an overall increase in microbial diversity compared to healthy controls (*p* < 0.05). Comparative abundance analysis identified 59 differentially abundant microbial species in non-metastatic pancreatic cancer (NMPC) (56 increased, 3 decreased) and 21 in metastatic pancreatic cancer (MPC) (19 increased, 2 decreased), alongside 18 significantly altered microbial metabolic pathways (FDR-adjusted *p* < 0.05). Notably, *Klebsiella pneumoniae*, *Klebsiella oxytoca*, and *Akkermansia muciniphila* were identified as prominent antibiotic resistance gene (ARG) carriers in the gut microbiota of PC patients, with 653 ARG subtypes detected across fecal samples, 38–47% of which were shared among groups. Strong co-occurrence patterns between ARGs (e.g., *acrB*, *mdtC*, *cpxA*, *emr*, *pmrF*) and the above species were observed predominantly in MPC samples (*p* < 0.05). Whole-genome sequencing of 14 isolates obtained from tumor tissue and pancreatic fluid revealed consistent ARG profiles and virulence genes, corroborating the metagenomic findings and supporting the hypothesis of gut-to-tumor translocation and potential intratumoral colonization.

**Conclusion:**

This study provides a comprehensive microbiome-based insight into PC and its metastatic subtypes. By integrating microbiome analysis with microbial culture, this study provides direct evidence of gut-derived multidrug-resistant (MDR) *K. pneumoniae* colonization in PC tissues.

## 1 Introduction

Pancreatic cancer (PC) was considered to be one of the most lethal malignant tumors of the digestive tract, with a five-year overall survival rate of less than 12% (Siegel et al., 2023). This prognosis worsened with the onset of metastasis, dropping survival rate to a mere 3% (Siegel et al., 2022). Despite advancements in multimodal diagnostic and treatment strategies, the efficacy of surgical interventions, which were crucial for effective PC management was significantly hindered by the challenges in the early detection (Vuijk et al., 2020). Metastasis was recognized as another main cause of the death of PC patients and treatment failure (Zheng et al., 2020). Although immunotherapy was demonstrated to be effective in a wide variety of metastatic cancers, it failed in PC because of its inherently “non-immunogenic” nature. Therefore, it is still necessary to explore new diagnosis and treatment strategies for PC (Li et al., 2023; Ni et al., 2023). The leading gene therapy and precision medicine strategies for PC, including KRAS-targeted interventions, miRNA-based treatments, and immunotherapies such as CAR-T, hold great promise yet face substantial challenges due to significant inter-individual variability in treatment response (Dwivedi et al., 2025; Sharma et al., 2025). Emerging evidence now identifies the gut microbiome, particularly through gut-to-tumor translocation, as a clinically detectable and individualized factor in PC progression and treatment resistance (Sethi et al., 2018; Kirsoy et al., 2024; Dwivedi et al., 2025).

The gut microbiota, residing within a complex and dynamic ecosystem, was closely linked to human immunity, primarily through its influence on intestinal permeability (Pushalkar et al., 2018). Contrary to prior beliefs of a sterile pancreas, emerging evidence suggests that gut microbiota can migrate to the pancreas via lymph nodes and dendritic cells, thereby facilitating the colonization of the pancreas by various microorganisms (Fan et al., 2018). This migration was implicated in the neoplastic transformation, with significant increases in the abundance of specific microbial taxa like *Proteobacteria*, *Synergistetes* and *Euryarchaeota* observed in PC patients (Thomas and Jobin, 2020). Additionally, early PC stages have been associated with alterations in microbiota’s metabolic pathways, notably in polyamine and nucleotide biosynthesis, establishing a link between these microbial processes and PC development (Mendez et al., 2020). Moreover, carbohydrates produced by *Malassezia* have been identified to stimulate PC cell growth through interaction with mannose-binding lectin, thereby initiating inflammatory immune responses (Zhong et al., 2023). Recent therapeutic strategies aimed at microbiota modulation, including fecal transplants and probiotic supplementation, have shown promise in offering new therapeutic benefits for PC patients, with fecal transplant therapies currently undergoing phase one clinical trials. The potential therapeutic impact of beneficial bacteria, such as *Aspergillus oryzae* and *Lactobacill*us, in inducing PC cell death and reducing gemcitabine drug resistance, brought new hope for cancer treatments (Chen et al., 2020).

In recent years, antibiotics have gradually been utilized to sensitize anti-tumor drugs and assist in prolonging the survival of cancer patients. However, the presence of antibiotic-resistant bacteria posed a significant challenge, potentially weakening antibiotic effectiveness and, in some cases, promoting tumor progression. Studies have shown that the survival rates of PC patients can be positively influenced by the application of quinolones in cases with high levels of *K. pneumoniae*, whereas resistance to these antibiotics can adversely affect patient outcomes (Konishi et al., 2021). The adjunct use of antibiotics, such as ciprofloxacin, has been observed to mitigate drug resistance, particularly against gemcitabine in colon cancer, highlighting the role of antibiotics in enhancing the response to immunosuppressive treatment (Mohindroo et al., 2021). The recent surge in extensive drug resistance and pan-drug resistance strains posed a significant global health challenge (Weniger et al., 2021), emphasizing the importance of identifying resistance genes for informed antibiotic selection in PC treatment. Despite the critical need, research into the resistome of tumor-associated bacteria remained limited. Moreover, few studies have validated the presence and genomic features of such tumor-associated bacteria using culture-based approaches combined with high-resolution sequencing. This study aims to systematically investigate the gut microbial composition and resistome in both metastatic and non-metastatic PC patients, identify potential microbial biomarkers and functional pathways, and provide evidence for the translocation and colonization of antibiotic-resistant *K. pneumoniae* from the gut to the tumor microenvironment through culture-based and genomic validation. By integrating metagenomic analysis of the fecal microbiome with whole-genome sequencing of tumor-derived isolates, this study seeks to investigate the microbial signatures associated with metastasis, chemoresistance, and tumor microenvironment modulation in PC.

## 2 Materials and methods

### 2.1 Ethical considerations

This investigation was conducted with strict adherence to ethical guidelines, ensuring informed consent and voluntary participation from all involved. The study cohort comprised 24 participants, including 13 pancreatic cancer (PC) patients, 1 intraductal papillary mucinous neoplasm (IPMN) patient, and 10 cohabitating spouses or family members of the PC patients. The study was conducted according to the guidelines of the Declaration of Helsinki, and approved by the Medical Science Research Ethics Committee of the First Hospital of China Medical University (protocol code [2022] 329 in July 2022). Informed consent was obtained from all subjects involved in the study.

### 2.2 Participant recruitment and sample collection

For metagenomic study, ten PC patients with a confirmed PC diagnosis with surgery and pathology were recruited between 2022 and 2023 from the First Hospital of China Medical University in Shenyang (**Table 1**). This included 5 patients with metastases (Group MPC: A1-A5) and 5 without (Group NMPC: B1-B5). Group MPC, A1, A4, and A5 were identified as those who experienced rapid recurrence within three months following surgery. 10 healthy controls (HCs), matched on age, gender, lifestyle, and dietary habits, were selected from the patient’s immediate circles (CA1-CA5 and CB1-CB3, CB5), with one exception where a patient’s daughter served as the control (CB4). To minimize inter-individual variability and control for lifestyle-related confounding factors, controls were selected from the patients’ immediate social networks (household members or close contacts). This matching strategy was chosen to ensure that the control group shared similar dietary habits, living environment, and healthcare access with the PC patients. Although this approach may reduce between-group differences, it maximizes the likelihood of identifying microbial signatures and antibiotic resistance gene (ARG) profiles specifically associated with PC rather than external exposures. Exclusion criteria encompassed recent antibiotic use within 3 months, probiotic or antifungal use within 1 month, significant physical impairments, and the presence of postoperative infections. The control group was screened according to the same fundamental criteria applied to PC patients, with the added stipulation of a minimum six-month antibiotic-free period. Fecal samples were collected 8 weeks after the discontinuation of antibiotics, with antibiotic use restricted to a maximum of 5 days, in postoperative patients. One patient (A3) in the MPC group did not undergo surgery; thus, samples were collected prior to the initiation of treatment. All samples were prepared in duplicate for analytical and backup purposes, and stored at −80 °C in sterile tubes.

**Table 1:** Clinical and demographic features of PCs and HCs.

Parameters	MPC and paired HCs		NMPC and paired HCs	
	PC patient (*n* = 5)	Control (*n* = 5)	PC patient (*n* = 5)	Control (*n* = 5)
Age, years				
< 65	3	3	2	3
≥65	2	2	3	2
Gender				
Female	2	3	2	4
Male	3	2	3	1
Tumor status				
T1	0		1	
T2	1		4	
T3	3		0	
T4	1		0	
Nodal status				
N0	3		5	
N1	2		0	
Metastatic status				
M0	3		5	
M1	2		0	
Pathologic status				
Stage I A	0		1	
Stage I B	1		4	
Stage II A	1		0	
Stage II B	1		0	
Stage III	0		0	
Stage IV	2		0	
Pancreatic tumor site				
Head	2		1	
Body	2		3	
Tail	1		1	
Sites of metastasis				
Liver	4		0	
Lung	1		0	
Others	0		0	
Surgical treatment				
PD	1		1	
DPS	2		4	
ERBD	1		0	
Non-Surgical Treatment	1		0	
Pathology				
Pancreatic ductal adenocarcinoma	5		5	
Smoking	1	2	1	0
Alcohol	1	0	0	0
Diabetes	0	0	1	0
Metformin	0	1	1	1
Obesity	0	0	0	0
Gastrointestinal Disease	0	0	0	0
ECOG PS				
0	1		4	
1	4		1	
2	0		0	
3	0		0	
4	0		0	
5	0		0	
BMI	20.02		24.16	
TBIL (umol/L)	64.2		21.2	
DBlL (umol/L)	49.4		6.8	
TC (mmol/L)	4.8		5.08	
CA19-9 (U/mL)	355.85		248.49	
CEA (U/mL)	3.86		3.44	
CA12-5 (U/mL)	17.25		17.32	
CA15-3 (U/mL)	17.22		10.352	
CA72-4 (U/mL)	5.11		3.186	

PC, Pancreatic cancer; PD, Pancreaticoduodenectomy; DPS, Distal pancreatectomy and splenectomy; ERBD, Endoscopic retrograde biliary drainage; ECOG PS, Eastern Cooperative Oncology Group Performance Status; TC, Total cholesterol; DBIL, Direct bilirubin; TBIL, Total bilirubin. Values of BMI, TBIL, DBIL, TC, CA19-9, CEA, CA12-5, CA15-3, CA72–4 were expressed as the mean ± SD. TNM, UICC/AJCC TNM staging for pancreatic cancer (8th ed., 2017).

For bacterial culture and following third-generation sequencing, pancreatic tumor tissue and pancreatic fluid were collected intraoperatively from four patients diagnosed with PC and IPMN under approved institutional ethical guidelines. Tissue homogenates and fluid samples were inoculated onto Columbia blood agar plates and incubated at 37°C for 18–24 hours under aerobic conditions. Control samples included operating room and non-tumorous surgical materials.

### 2.3 Metagenomic study for fecal samples

#### 2.3.1 DNA extraction and metagenomic sequencing

Genomic DNA was extracted using the TIANamp Stool DNA Kit as per the manufacturer’s guidelines, with its concentration and purity verified through agarose gel electrophoresis and UV absorbance measurements (NanoDrop ND1000). Sequencing libraries were prepared using the Illumina TruSeq^®^ DNA PCR-Free Sample Preparation Kit, assessed for quality, and sequenced on an Illumina platform to achieve 150 bp paired-end reads, which were filtered based on quality score (minimum Q30 for 90% of bases), removal of adapter sequences, and minimum read length (≥50 bp), generating approximately 10 GB of clean data per sample. The clean data were evaluated for sequencing quality using MultiQC. Data output statistics for both the raw and cleaned data were generated using ReSeqTools. Further, host-derived reads were removed by aligning sequences against the human genome using BMTagger, as recommended by NCBI.

#### 2.3.2 Microbiome characterization

Kraken2 (Simpson et al., 2021) was used to process all the metagenomic sequencing data and Bracken (Lu et al., 2017) was for correction. A cladogram was produced by GraPhlAn (Wood et al., 2019). HUMAnN3 (Franzosa et al., 2018) (nucleotide-database: chocophlan; protein-database: uniref 90) software was performed to determine microbial pathways and abundances.

#### 2.3.3 Metagenome assembly and identification of ARGs

Assigned based on specific barcode and primer sequences, paired-end reads underwent stringent quality control. High-quality reads were assembled using MEGAHIT, with open reading frames predicted by MetaGeneMark and subsequently clustered to minimize redundancy. The assembled genomes were evaluated for completeness and contamination, with taxonomic profiling of open reading frames conducted via DIAMOND against a comprehensive microbial database. ARG sequences were classified into more than 20 categories using data from the Comprehensive Antibiotic Resistance Database (CARD), followed by taxonomic assignment of ARG-carrying contigs using Kraken2. Host-ARG associations were further refined using stringent thresholds and strong correlation analyses, including sequence coverage (≥90%) and identity (≥95%). Additionally, the identified ARGs were cross-checked against two public databases: NT (Nucleotide Sequence Database) and RefSeq (NCBI Reference Sequence Database).

### 2.4 Isolation and characterization of bacteria from PC tissue and pancreatic fluid

#### 2.4.1 Whole genome sequencing and assembly

To validate the presence of *K. pneumoniae* identified via metagenomics, we employed aerobic culture using blood agar medium, guided by the taxonomic and resistance profiles derived from fecal sequencing, a strategy focused on this specific facultative pathogen rather than on broad microbial diversity. Given that *K. pneumoniae* was the most prominent species enriched in PC patients and showed strong ARG associations, the culture conditions were specifically optimized for its isolation and downstream genomic confirmation. A total of 14 K*. pneumoniae* isolates from tumor tissue (PANC strains) and pancreatic fluid (PANF strains) were subjected to whole-genome sequencing using the Oxford Nanopore MinION platform. Genomic DNA was extracted using a QIAamp DNA Mini Kit (Qiagen) and quantified using a Qubit fluorometer. Libraries were prepared with the Rapid Barcoding Kit (Oxford Nanopore Technologies) and sequenced with R9.4.1 flow cells. Base calling was performed using Guppy, and reads were assembled *de novo* with Flye (v2.9).

#### 2.4.2 Genome annotation and functional gene prediction

The assembled contigs were annotated using ABRicate (v1.0.1). ARGs were identified using the CARD (Comprehensive Antibiotic Resistance Database), while virulence factors were annotated against the VFDB (Virulence Factors Database). Genomic islands and gene clusters of interest were visualized on circular genome plots using CGView.

#### 2.4.3 Phylogenetic analysis and comparative genomics

To investigate evolutionary relationships among isolates, core genome alignments were generated using Roary and multiple sequence alignments were performed with MAFFT. A maximum likelihood phylogenetic tree was constructed using FastTree and visualized with iTOL. Tree topologies were annotated with presence/absence heatmaps for virulence and resistance genes, as well as predicted antibiotic resistance phenotypes.

### 2.5 Statistical analysis

Alpha diversity was quantified using the Shannon index and analyzed with R software, employing the Wilcox.test for between-group comparisons. Metastats facilitated the identification of significant taxonomic differences, while network analysis, supported by Pearson’s rank correlations in Cytoscape, elucidated microbial and ARGs co-occurrence patterns. Significance was established at a *p*-value threshold of <0.05. To compare the species differences of group PC with group HC, categorical data were tested using the”edgeR”package (Pereira et al., 2018) (calcNormFactors: trimmed mean of M-values method). The statistical analysis for differentially expressed (DE) was done using edgeR (glmLRT test). Significant differences of in functional pathways among groups were determined by performing the”EasyAovWlxPlot.R” package. The microbial community heatmap was clustered and visualized by the “ComplexHeatmap” package. To account for potential false positives arising from multiple hypothesis testing, we have applied false discovery rate (FDR) corrections using the Benjamini-Hochberg procedure throughout the study. The Spearman rank correlation coefficient was used to evaluate the correlation between phenotypes and the correlation between microbiome features. Correlations with corresponding empirical *p*-values less than 0.05 were retained.

## 3 Results

### 3.1 Illumina sequencing read statistics

Illumina sequencing generated on average 11.6 GB of base-called data across 20 metagenomics libraries (**Supplementary Table S1**). The sequencing depth was verified to be adequate, as indicated by the stability of rarefaction curves using the Shannon index, observed species, and the Chao1 estimator (**Supplementary Figure S1**). Approximately 91.2% of bases across all samples achieved an average Phred score of Q30 or above, indicating high-quality of nucleobase that generated by DNA. A total 1,561,203 high-quality (length > 500bp) assembled contigs were generated from all 20 samples with a range of 23,678–154,881 sequences per sample (**Supplementary Table S2**). Regarding the contigs number and length of all samples, no significant differences were observed between cases and controls either between the MPC group NMPC group.

### 3.2 Alterations of microbiome communities

**Figure 1:**
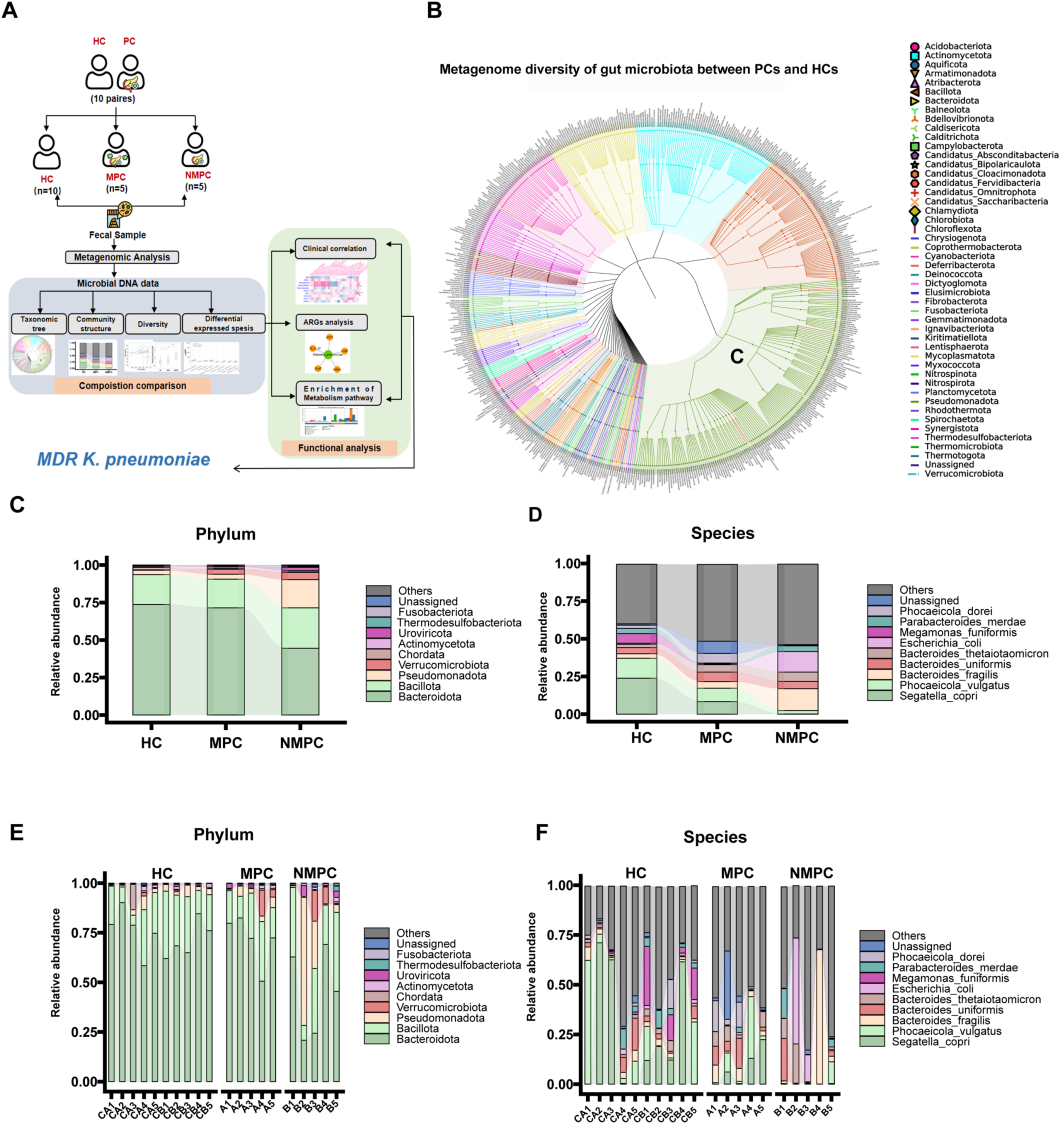
Compositional analysis of bacterial gut microbiota. **(A)** Metagenomic study design process diagram. **(B)** The taxonomic tree for different samples at genus taxonomic level. Different taxonomic type was represented by different color of nodes. **(C, D)** Different compositions of gut microbiota from HC, MPC and NMPC groups’ fecal sample at the phylum level **(C)**, at the genus level **(D)**. **(E, F)** The corresponding microbial composition analysis of each sample and relative frequency of the top 10 at the phylum level **(E)**, at the genus level **(F)**. Certain icons used in subfigure A were obtained from Flaticon (https://www.flaticon.com) under proper license.

To investigate the differences in gut microbiota between PC and the paired HC group, a metagenomic analysis was conducted. The whole bacterial diversity of the gut microbiota in PC and HC groups was shown in **Figure 1B**. Dividing the PC group into NMPC and MPC subgroups, we observed distinct microbial communities. The top ten phylum and genus of bacteria (**Figures 1C, D**) particularly revealed differences at the genus levels between NMPC, MPC and HC groups. Dominant genera such as *Bacteroides fragilis*, *Escherichia coli* and *Parabacteroides merdae* in NMPC fecal samples, and *Phoceaicola dorei*, *Bacteroides uniformis* and *Bacteroides thetaiotaomicron* in MPC fecal samples were particularly contrasting with those in HC fecal samples (**Figures 1E, F**).

### 3.3 Analysis of microbiome diversity

**Figure 2:**
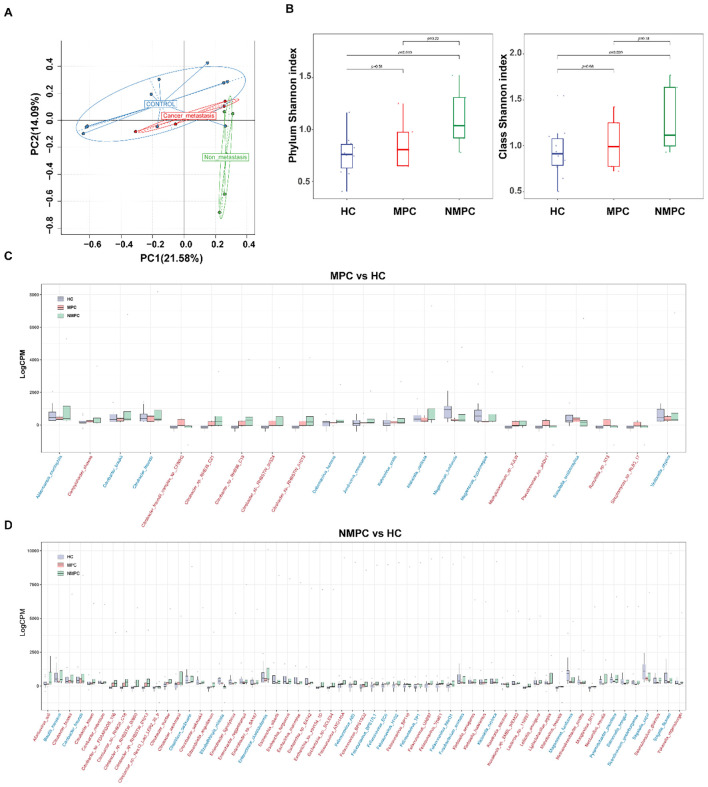
Changes of gut microbiota in PC patients. **(A)** Principal component analysis (PCA) of gut microbiota profiles (species level) from NMPC, MPC, and HC groups. The first two principal components (PC1 and PC2) explain ~36% of the total variance across samples and reflect the major axes of microbial community differentiation. **(B)** Alpha diversity assessed by the Shannon index across NMPC, MPC, and HC groups. Boxplots show medians and interquartile ranges; statistical significance determined by Wilcoxon test. **(C, D)** Differentially abundant bacterial species in fecal samples from MPC (C, n = 21 species) and NMPC (D, n = 59 species) compared to HC, identified using the edgeR package (glmLRT test, p < 0.05). Each dot represents a sample’s relative abundance. Horizontal axes list species names, with red labels indicating increased abundance and blue labels indicating decreased abundance in MPC or NMPC versus HC. Vertical axes show normalized relative abundance. Horizontal lines denote the median values across samples in each group.

Extending our examination to the role of the microbiome in PC patients, principal coordinate analysis was utilized to assess beta diversity. Our results indicated distinct clustering within the NMPC group, in contrast to the MPC and HC groups (**Figure 2A**). Then alpha diversity at the phylum and class level were conducted for diversity comparison. The Shannon index value for the NMPC group was significantly higher than that for the HC group at phylum level, indicating increased bacterial diversity in fecal samples of the NMPC than in the HC group. No significant differences were observed between MPC and HC groups (**Figure 2B**). These data suggested enhanced microbial gut microbiota diversity within gut microbiota of PC patients, particularly in NMPC subgroup.

### 3.4 Changes of species abundance

**Figure 3:**
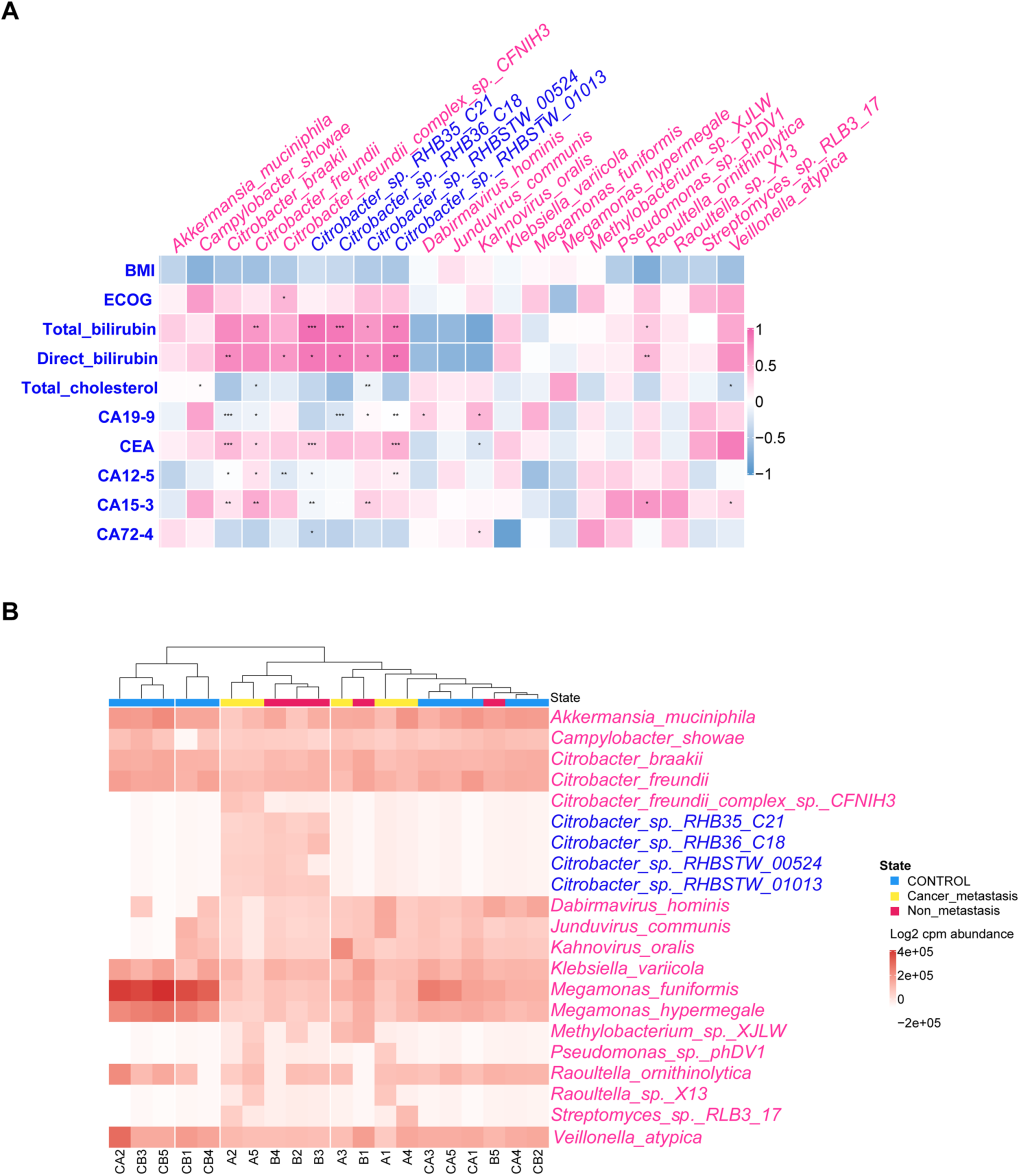
Correlation between dominant species and clinical features of PC patients. **(A)** Correlation of relative abundance of differentially expressed bacterial species and PC’s clinical features by Spearman’s rank correlation. The pink and blue heat map representing positive and negative correlation, respectively, *p < 0.05, **p < 0.01, ***p < 0.001. **(B)** Microbial community heatmap with cluster analysis, and the color intensity in each grid showed the percentage in a sample, referring to color key at the right.

Upon examining changes of relative abundance in bacterial community, we noted distinct patterns between MPC (**Figure 2C**) and NMPC (**Figure 2D**) when compared to HC group. Significant differences in 21 species were observed between MPC and HCs, with 19 increased and 2 decreased species. Conversely, the NMPC and HC comparison revealed 59 species with significant abundance shifts, with 56 increased and 3 decreased species. Notably, both MPC and NMPC groups exhibited significant depletion of *Megamonas funiformis*, known for its probiotic properties. Moreover, the presence of *Felixounavirus, Citrobacter, Klebsiella, Escherichia and Raoultella* was most significantly linked to PC. The normalized abundance of dominant species in the gut microbiota across samples was shown as a heatmap (*p* < 0.05) in **Figure 3A**. Correlations between dominant species and clinical features of PC patients were further explored. The results included the strong and positive correlation of the genus *Citrobacter* (especially *Citrobacter* sp. *RHB35_C21*, *Citrobacter* sp. *RHB36_C18 and Citrobacter* sp. *RHBSTW 01013*) with both total and direct bilirubin levels, however, a relatively weak and positive correlation with CEA in blood. It’s also worthy to mention that the significant correlations were observed between PC biomarkers of CEA, CA12-5, CA15–3 and *Citrobacter freundii* or *Citrobacter braakii* species. A microbial community heatmap with cluster analysis, and the color intensity in each grid shows the percentage in a sample, referring to the color key at the right (**Figure 3B**). Microbial genus *Citrobacte*r was significantly clustered in the PC group. These results suggested the potential role of the differential bacterial species within the gut microbiota in the diagnosis of PC patients.

### 3.5 Functional enrichment analysis of microbial metabolic pathways

**Figure 4:**
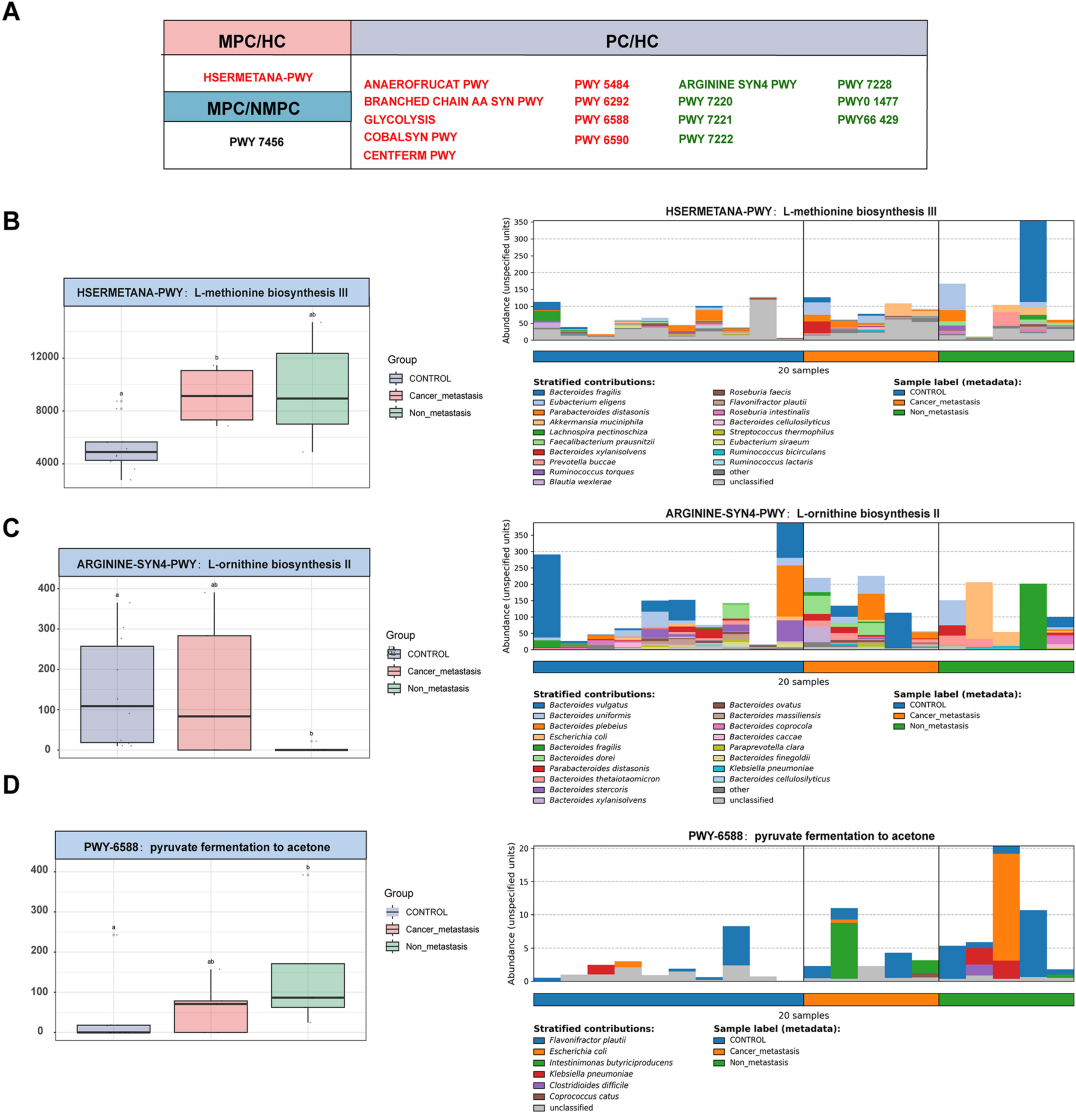
Enrichment of microbial metabolic pathways. **(A)** Significant alterations of 18 metabolic pathway of each sample among gut microbiota in MPC, NMPC group and paired HCs. Red color represented a significant increase of microbial abundance compared to HC, while green color represented a decrease. Stratified contributions of the microbial pathways of **(B)** HSERMETANA-PWY, **(C)** ARGININE_SYN4 PWY and **(D)** PWY 6588 pyruvate were showed as examples.

We further explored the enrichment of functional pathways in microbial metabolism. Analysis of metagenomic data identified 18 significantly altered metabolic pathways (*p* < 0.05, **Figure 4A**), with the MPC group showing a significant increase in HSERMETANA-PWY compared to the HC group, and in PWY 7456 compared to the NMPC group. Among the pathways showing significant changes in the NMPC group, there were 9 increased pathways and 7 decreased pathways. The classification of functional pathways as amino acid (ARGININE SYN4 PWY, BRANCHED CHAIN AA SYN PWY, PWY 6292, COBALSYN PWY, HSERMETANA PWY), carbohydrate (PWY 6588, CENTFERM PWY, GLYCOLYSIS, PWY 5484, ANAEROFRUCAT PWY, PWY 6590), nucleotide metabolism (PWY 7220, 7221, 7222, 7228), and fatty acid (PWY66 429, PWY0 1477) were summarized in **Table 2**. Their corresponding stratified contributions analysis of HSERMETANA-PWY (**Figure 4B**), ARGININE SYN4 PWY (**Figure 4C**) and PWY 6588 pyruvate (**Figure 4D**) and other pathways (**Supplementary Figure S2**) also demonstrated the significant metabolic alterations among MPC and NMPC patients.

**Table 2:** Enriched 18 differently expressed metabolic pathways in HC, MPC and NMPC groups.

Classifications	ID	Description
Amino acid metabolism	ARGININE SYN4 PWY	L ornithine biosynthesis II g *Bacteroides.s Bacteroides ovatus*
	BRANCHED CHAIN AA SYN PWY	superpathway of branched chain amino acid biosynthesis g *Flavonifractor.s Flavonifractor plautii*
	PWY 6292	superpathway of L cysteine biosynthesis (mammalian)
	COBALSYN PWY	superpathway of adenosylcobalamin salvage from cobinamide I
	**^#^**HSERMETANA PWY	L methionine biosynthesis III
Nucleotide metabolism	PWY 7220	adenosine deoxyribonucleotides *de novo* biosynthesis II g *Bacteroides.s Bacteroides ovatus*
	PWY 7221	guanosine ribonucleotides *de novo* biosynthesis g *Bacteroides.s Bacteroides ovatus*
	PWY 7222	guanosine deoxyribonucleotides *de novo* biosynthesis II g *Bacteroides.s Bacteroides ovatus*
	PWY 7228	superpathway of guanosine nucleotides *de novo* biosynthesis I g *Bacteroides.s Bacteroides ovatus*
Carbohydrate metabolism	PWY 6588	pyruvate fermentation to acetone g *Flavonifractor.s Flavonifractor plautii*
	CENTFERM PWY	pyruvate fermentation to butanoate
	GLYCOLYSIS	from glucose 6 phosphate
	PWY 5484	glycolysis II (from fructose 6 phosphate)
	ANAEROFRUCAT PWY	homolactic fermentation
	PWY 6590	superpathway of *Clostridium acetobutylicum* acidogenic fermentation
	^*^PWY 7456	6 β (1,4) mannan degradation
Fatty Acid metabolism	PWY66 429	fatty acid biosynthesis initiation (mitochondria) g *Bacteroides.s Bacteroides ovatus*
	PWY0 1477	ethanolamine utilization

MPC, Metastatic pancreatic cancer; NMPC, Non-metastatic pancreatic cancer. Note, microbial pathways and abundance were determined by HUMAnN3. The statistical analyses were performed by “EasyAovWlxPlot.R” package. ID names that marked with *represented the comparison between MPC and NMPC, while **^#^** represented the comparison between MPC and NMPC, others were the comparison between NMPC and HC.

### 3.6 Occurrence of gut microbial ARG profiles

**Figure 5:**
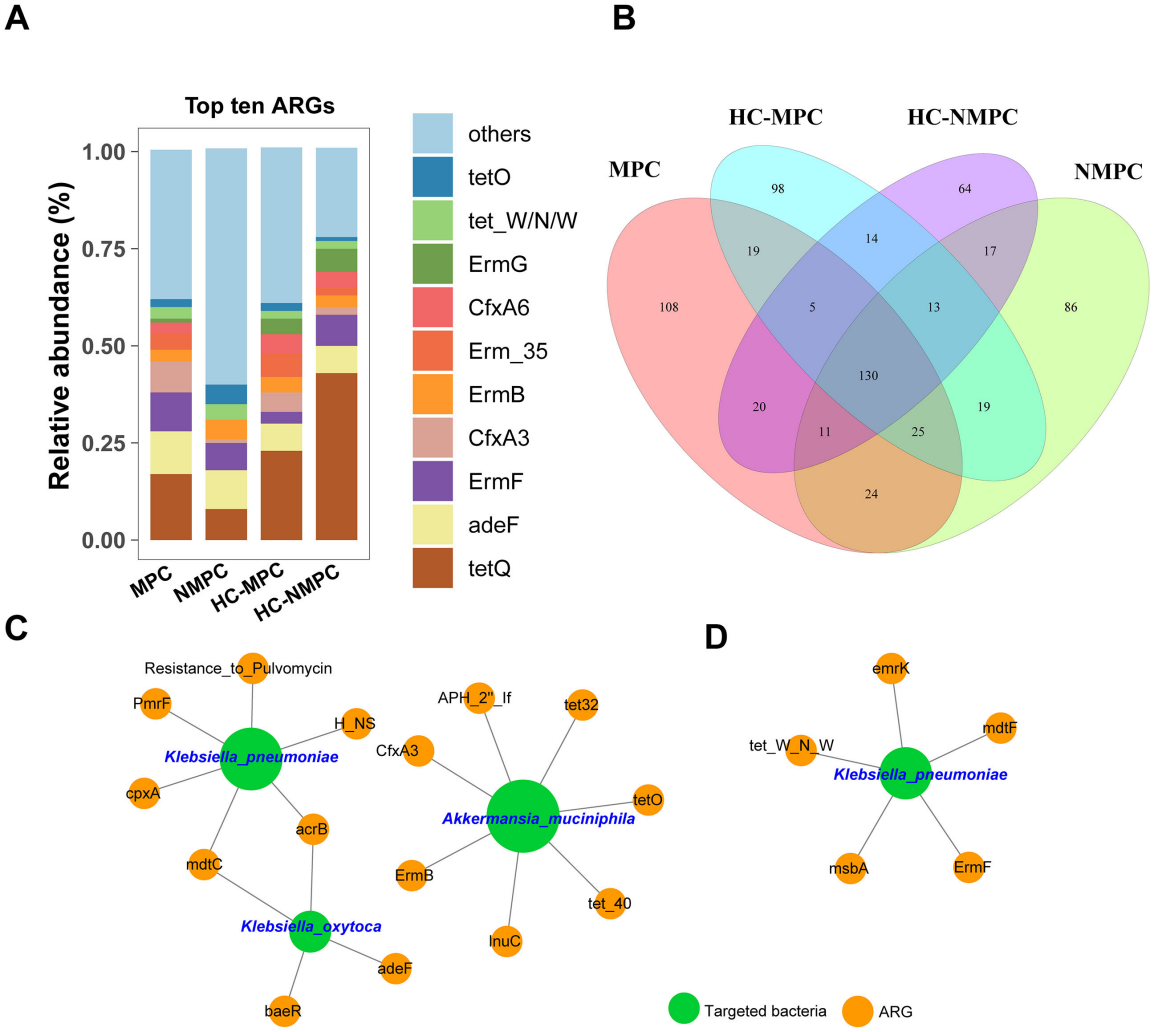
Profiling and network analysis of antibiotic resistance genes (ARGs) in fecal samples from different participant groups. **(A)** Stacked bar chart showing the relative abundance of the top 10 dominant ARG subtypes across four groups. **(B)** Venn diagram depicting the distribution of shared and distinct ARG subtypes within the samples of four groups. **(C, D)** representing the network analysis between the targeted bacterial species and ARGs conducted in fecal samples of group MPC and NMPC, respectively. The charts have specifically been drawn to highlight which ARGs and targeted bacteria are actually linked. A connection represents an extremely strong (Pearson’s r >0.9) and significant (*p* < 0.001) correlation. The nodes with green and orange colors represent targeted bacteria and ARGs, respectively. The size of each node is proportional to the number of connections between nodes.

A total of 653 ARG subtypes conferring resistance to 37 different antibiotic classes were detected across all fecal samples, with 130 overlapping ARGs present in all samples, accounting for 38%-47% of ARGs detected in each group (**Figure 5A**). The gene tetQ, encoding resistance to tetracycline, was the most common across samples, except in group NMPC (**Figure 5B**). Resistance genes to tetracycline had the highest frequency in all sample types, especially in HC group samples. In addition to *tet*Q, *ade*F (the multidrug efflux transporter) and macrolide resistance genes (*Erm*B and *Erm*F) were also prevalent (**Figure 5B**). The detected ARG subtypes represent major resistance mechanisms, including cellular protection, antibiotic inactivation, efflux pumps, and antibiotic target alteration.

### 3.7 Co-occurrence pattern between targeted bacteria and ARGs

Network analysis was employed to investigate co-occurrence patterns between microbial taxa and ARG subtypes. Three bacterial species were identified as probable ARG hosts based on co-occurrence analysis results (**Figures 5C, D**), specifically *K. pneumoniae*, *Klebsiella oxytoca*, and *A.muciniphila*, all of which have been implicated in the differentially expressed species of PC patients. Notably, these strong connections were observed exclusively in samples from the NMPC group, with stronger correlations in samples from PC with metastases (**Figure 5C**) and *K. pneumoniae*, *K. oxytoca*, and *A. muciniphila*, all of which have been implicated in the differentially expressed species of PC patients. Notably, these strong connections were observed exclusively in samples from the PC group, with stronger correlations in samples from PC with metastases (**Figure 5C**, **Supplementary Table S3**) compared to those without metastases (**Figure 5D**, **Supplementary Table S4**). Both *K. pneumoniae* and *K. oxytoca*, classified as *Gammaproteobacteria*, commonly carried the *Mdt*C and *Arc*B genes, associated with multidrug resistance and the regulation of detoxification-related genes, respectively. *K. pneumoniae* was also a potential host for the polymyxin resistance gene (*pmr*F) and an efflux pump pump gene (*cpx*A), whereas *A. muciniphila* harbored up to seven ARGs, including those conferring resistance to tetracycline (*tet*32, *tet*40, *tet*O), MLS (*erm*B), β-lactam (*cfx*A3), and lincomycin (*lnu*C). Moreover, *K. pneumoniae* was associated with five ARG subtypes, including resistance genes for tetracycline (*tet*_W_N_W), MLS (*erm*F), and multidrugs (*mdt*F, *msb*A, and *emr*K). Finally, we filtered and validated the ARGs carried by the targeted host bacteria, including *K. pneumoniae*, *Klebsiella oxytoca*, and *A.muciniphila.* The results were presented in **Supplementary Table S5**, with associations between ARGs and host bacteria in samples from PC patients highlighted in bold for clarity.

### 3.8 Isolation and identification of *K. pneumoniae* from PC tissue and pancreatic fluid

**Figure 6:**
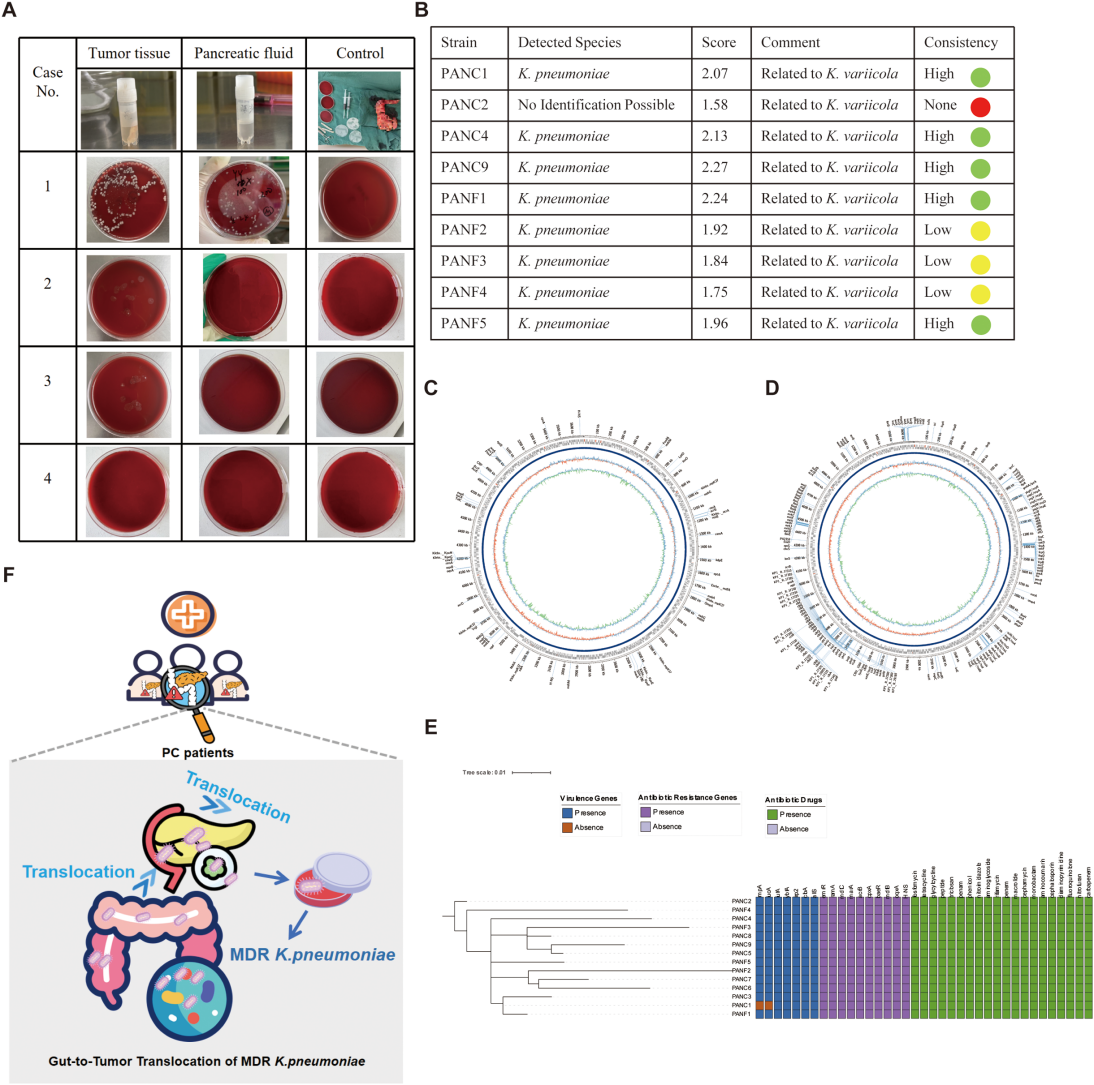
Isolation, identification, genomic and functional profiling of *K*. *pneumoniae* from pancreatic tumor tissue and pancreatic fluid. **(A)** Blood agar culture results of tumor tissue and pancreatic fluid from four patients with pancreatic cancer. Among them, only Case 1 yielded colonies from both tumor tissue and pancreatic fluid, while the other three cases showed no visible growth. Controls from operating room remained sterile. **(B)** MALDI-TOF MS (Matrix-Assisted Laser Desorption/Ionization Time-of-Flight Mass Spectrometry) identification of bacterial isolates. Eight strains were confirmed as *K*. *pneumoniae* with high identification scores and high consistency, and most were closely related to *K*. *variicola*, indicating potential phylogenetic affiliation. **(C–D)** Circular genome maps of one *K*. *pneumoniae* strain (PANC1 from tumor tissue) generated using Oxford Nanopore long-read sequencing. The assembled genome was annotated using ABRicate with two different databases: CARD for **(C)** and VFDB (Virulence Factors Database) for virulence genes **(D)**. The outermost rings display coding sequences (CDSs) on both DNA strands, with annotated resistance and virulence genes labeled. The inner rings represent GC content (green/red) and GC skew (purple/blue). Resistance genes and virulence genes are prominently marked, illustrating the multidrug-resistant and hypervirulent potential of the strain. **(E)** Phylogenetic tree and heatmap of 14 K*. pneumoniae* strains (PANC, from tumor tissue; PANF, from pancreatic fluid) based on whole genome sequencing. The left dendrogram shows genomic relationships, while heatmaps to the right illustrate the presence (colored) or absence (blank) of virulence genes (blue), antibiotic resistance genes (purple), and corresponding antibiotic resistance phenotypes (green). The PANC1 and PANF1 strains from the same patient clustered closely, suggesting a common origin. The figure highlights diverse resistance and virulence profiles among the isolates. **(F)** Schematic illustration of the translocation of MDR *K*. *pneumoniae* from the gut to pancreatic tumor tissue. Certain icons used in subfigure A were obtained from Flaticon (https://www.flaticon.com) under proper license.

In the initial stage of this study, fecal metagenomic sequencing of PC patients revealed a strong positive correlation between *K. pneumoniae* and a broad array of ARGs, including key efflux pump-related genes such as *acrB*, *mdtC*, *cpxA*, *baeR*, and *H-NS*. These genes are known to play critical roles in multidrug resistance mechanisms and suggested a potential for stable intestinal colonization and resistance development. To validate these findings and assess possible bacterial translocation beyond the gut, we collected tumor tissue and pancreatic fluid samples intraoperatively from 3 PC patients (**Table 3**). Additionally, samples obtained from a patient with IPMN were utilized as negative controls to ensure the specificity of our observations. Blood agar culturing revealed that in one patient (Case 1), *K. pneumoniae* was successfully isolated from both tumor tissue and pancreatic fluid (**Figure 6A**). Mass spectrometry-based species identification confirmed high-confidence hits of *K. pneumoniae* in both compartments (PANC1 and PANF1), while no bacterial growth was observed in the control group or other patients (**Figure 6B**). This result suggested that *K. pneumoniae* may colonize the tumor-associated microenvironment or migrate retrogradely through the pancreatic duct system.

**Table 3:** Clinical and demographic features of PC and IPMN patients.

Parameters	PC patient (Case 1)	PC patient (Case 2)	PC patient (Case 3)	IPMN patient (Case 4)
Age	76	72	76	60
Gender	Male	Female	Male	Male
Pathologic status	Stage II B	Stage II B	Stage II A	–
Pathological Lesion Site	The pancreatic head	The pancreatic head	The pancreatic body-tail	The pancreatic body-tail
Tumor status	T2	T2	T3	–
Nodal status	N1	N1	N0	–
Metastatic status	M0	M0	M0	–
Surgical Treatment	PD	PD	DPS	DPS

PC, Pancreatic cancer; IPMN, Intraductal papillary mucinous neoplasm;PD, Pancreaticoduodenectomy; DPS, Distal pancreatectomy and splenectomy.

### 3.9 Whole-genome sequencing confirms ARG and virulence profiles consistent with fecal metagenomics

To further characterize the genetic basis of these strains, we performed whole-genome sequencing (WGS) of 14 *K. pneumoniae* isolates using Oxford Nanopore long-read technology. The assembled circular genomes of PANC1 and PANF1 demonstrated conserved genomic architecture with rich annotations of both ARGs and virulence factors (**Figures 6C, D**). Notably, long-read WGS confirmed the presence of several resistance determinants previously identified in fecal metagenomes, including *acrB*, *mdtC*, *cpxA*, *baeR*, *H-NS*, and multiple *emr*-family efflux genes (*emrK*, *emrD*, etc.), validating the consistency of ARG profiles across intestinal and extraintestinal compartments. In addition, core genome phylogenetic analysis (**Figure 6E**, left) revealed high genomic similarity between tumor- and fluid-derived isolates, supporting a common clonal origin or intra-host migration. The heatmap summary (**Figure 6E**, right) showed that nearly all strains carried classic hypervirulence genes (*rmpA*, *iucA*, *ybtA*, *wzi*) and a wide spectrum of ARGs, including *blaSHV-190*, *oqxA/B*, and *fosA*, as well as resistance to aminoglycosides, cephalosporins, and carbapenems. Taken together, these results suggest that *K. pneumoniae* can translocate from the gut to tumor tissues and pancreatic fluid, maintaining its MDR and virulent nature. The convergence of metagenomic and isolate-based ARG detection underscores the potential clinical risk posed by intratumoral colonization of MDR *K. pneumoniae*, which may impair the efficacy of chemotherapeutic agents such as gemcitabine and increase the likelihood of treatment resistance.

## 4 Discussion

**Figure 7:**
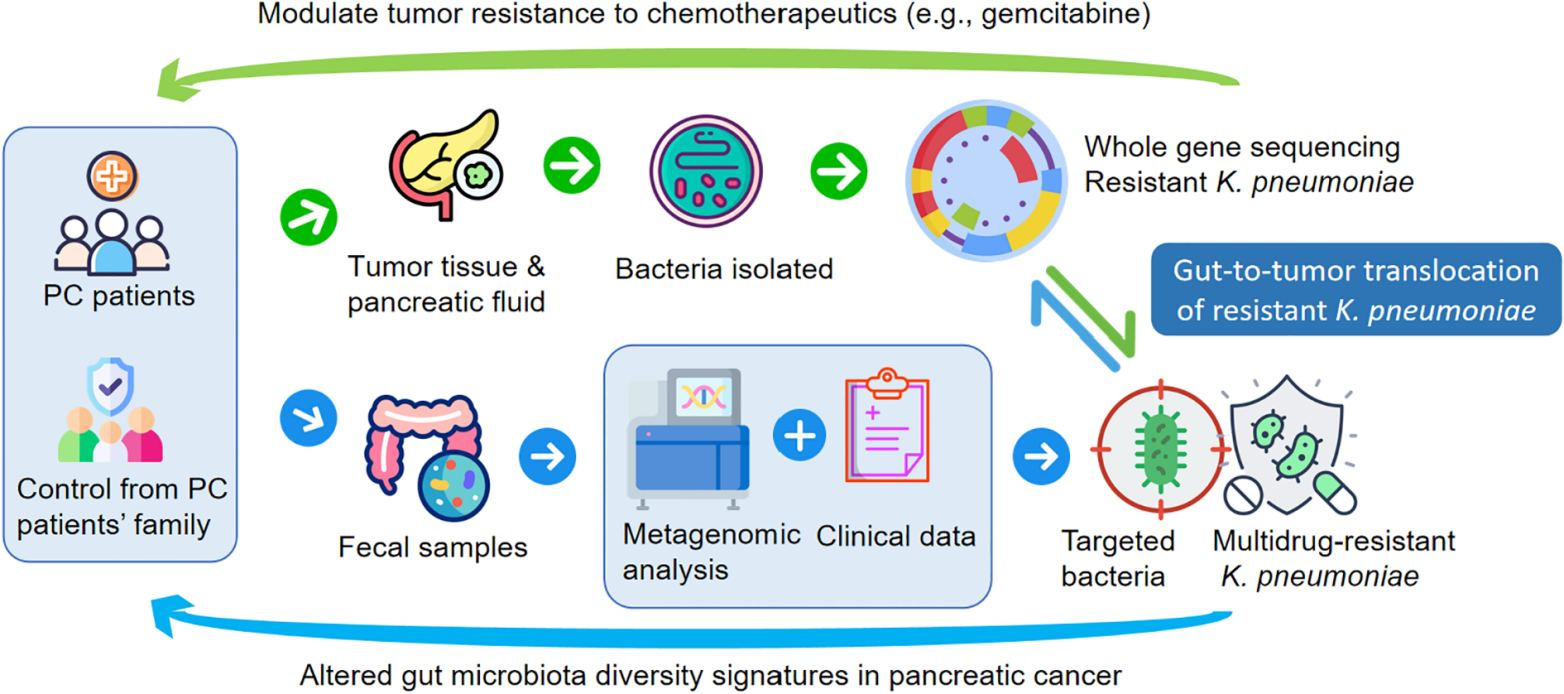
Certain icons used in subfigure A were obtained from Flaticon (https://www.flaticon.com) under proper license.

Pancreatic cancer (PC) remains one of the deadliest malignancies in the digestive tract, with limited survival improvements despite advances in precision oncology. Although surgery remains the most effective treatment for PC, postoperative recurrence and metastasis remain urgent clinical challenges. The absence of precise biomarkers and specific early symptoms contributes to delayed diagnosis and poor prognosis. While smoking, alcohol consumption, type 2 diabetes, chronic pancreatitis, and familial genetic predisposition are recognized as key risk factors for PC (Rawla et al., 2019), many patients develop the disease without presenting these feature. Recent evidence has highlighted the influence of host-associated microbial communities on PC development, suggesting their potential as early diagnostic markers for PC (Riquelme et al., 2019). In this study, we systematically analyzed gut microbiota signatures in metastatic and non-metastatic PC patients through metagenomic profiling. Given the impact of host environment on microbiota (Lao et al., 2024), we included matched HCs to control for inter-individual variation. The strict inclusion criteria and pair-matched design, while constraining the sample size, were instrumental in identifying a reliable microbial signature with minimal confounding. The agreement between computational and culture-based evidence not only confirms the gut-to-tumor translocation of MDR (**Figure 7**). Although aerobic culture but also provides compelling biological validation for this process. Therefore, this work should be regarded as a foundational study that provides a validated target and a methodological framework for subsequent large-scale validation. Our data revealed significantly increased microbial diversity in PC patients compared to HCs, with distinct community structures between NMPC and MPC.

Importantly, the MPC group included patients with confirmed metastasis and those who experienced rapid postoperative recurrence, suggesting that gut microbiota profiles may carry prognostic value. These results support previous studies linking microbiota to PC progression (Liu et al., 2024). Specifically, 59 and 21 species showed significant differences between PC/HC and MPC/HC, respectively. We confirmed the depletion of *M. funiformis* (Zhou et al., 2021) in PC patients and observed a reduction in *Citrobacter freundii*, a species previously linked to methionine γ-lyase with potential anti-tumor effects (Kharofa et al., 2023). Interestingly, *Veillonella atypica*, previously reported in tumor and oral microbiota (Raboni et al., 2018), was significantly reduced in the gut of MPC patients. Additionally, pathogens such as *Edwardsiella anguillarum*, *Lelliottia amnigena*, *Klebsiella variicola*, and *Shigella flexneri* showed altered abundance, which has not been reported in PC before (McKinley et al., 2023). Microbiota signatures also revealed clinical associations that may provide functional insights. The infection-associated genera *Citrobacter* (Koeninger et al., 2021) and *Raoultella ornithinolytic*a (Seixas et al., 2021), both known gastrointestinal pathogens, were positively associated with elevated bilirubin levels in PC patients. These results further emphasized the interaction between gut-derived bacteria and systemic inflammation in PC. Our analysis also revealed significant alterations in 18 metabolic pathways in PC patients, with notable contributions from specific microbial species, particularly *A. muciniphila* and *K. pneumoniae*, to these metabolic changes. Of particular significance was the marked alteration of the L-methionine pathway in MPC patients. This observation was of substantial clinical relevance, as methionine has been well-documented to play crucial roles in both intestinal function and barrier integrity (Chen et al., 2014), as well as in tumor metastasis, including PC progression (Govaerts et al., 2021; He et al., 2022). These findings were further substantiated by emerging evidence demonstrating the pivotal role of gut microbiota in host methionine metabolism (Sanderson et al., 2019; Kaiser, 2020; Zhao and Lum, 2022).

Currently, the growing recognition of the role of pathogenic bacterial overgrowth within tumors in tumorigenesis and chemoresistance (Cruz et al., 2024).The intratumoral microbiota plays an emerging role in shaping chemotherapy response and tumor immune environment. *Gammaproteobacteria* in tumors have been shown to inactivate gemcitabine via cytidine deaminase isoforms (Sayin and Mitchell, 2023), reducing its efficacy. On the other hand, tryptophan-derived indole-3-acetic acid has been reported to amplify gemcitabine effects (Tintelnot et al., 2023). Another study of high ethanolamine (EA) levels not only proved to be associated with worse survival (Battini et al., 2017), but also proved to participate in *Klebsiella*’s adaptive drug resistance (Norsigian et al., 2019). Adjunctive antibiotics like quinolones have been proposed to overcome resistance (Weniger et al., 2021). However, the wide spread of ARGs among gut microbes may hinder these interventions. Our results further implicated *K. pneumoniae* as a central contributor to ARG burden and potential chemoresistance in PC. Both *K. pneumoniae* and *K.oxytoca* (*Gammaproteobacteria*) demonstrated significant enrichment in PC samples, particularly among patients in the MPC patients. Notably, these bacterial species exhibited strong associations with diverse antibiotic ARGs, with *K. pneumoniae* showing prominent correlation. Moreover, the intraoperative isolation of *MDR K. pneumoniae* from matched tumor and pancreatic fluid provides direct microbiological evidence for our central hypothesis of gut-to-tumor translocation, confirming its role as a clinically significant pathogen in PC. A previous study suggested bacteria class *Gammaproteobacteria* (e.g., *K. pneumoniae*) harbored in PC tumor was able to metabolize the gemcitabine to the inactive 2’, 2’-difluorodeoxyuridine through a long isoform of the enzyme cytidine deaminase (Ertz-Archambault et al., 2017; Geller et al., 2017; Sayin and Mitchell, 2023; Horvat et al., 2024). Moreover, bacteria within tumors can inactivate chemotherapeutic agents such as gemcitabine via bacterial enzymes like cytidine deaminase (CDD) (Pushalkar et al., 2018). Notably, ARGs such as *acrB*, *mdtC*, *cpxA*, *baeR*, *H-NS*, and emr genes were frequently co-localized with *K.pneumoniae*, suggesting potential roles in chemoresistance.

With an intact intestinal barrier, the pancreas and its alkaline secretions were historically considered a sterile environment. However, studies have identified the presence of intratumoral microbiota within PC tissues, which has been linked to PC outcomes (Riquelme et al., 2019; Abe et al., 2024). Moreover, the presence of gut microbiota in pancreatic tissues under normal conditions (Sammallahti et al., 2021) highlights the physiological relevance of the gut–pancreas axis (Ahuja et al., 2017). Although these results provide valuable insights, the role of ARGs within intratumoral microbiota in pancreatic tissues remains significantly understudied in current research literature. To directly validate gut–tumor translocation, we isolated *K. pneumoniae* strains from tumor and pancreatic fluid in PC patients (**Figure 6F**). Out of four patients sampled, one yielded positive cultures from both tumor tissue and pancreatic fluid; 14 isolates underwent Nanopore sequencing. Although aerobic culture on a single medium may limit the detection of obligate anaerobes or fastidious organisms, our approach was hypothesis-driven: based on metagenomic findings indicating *K. pneumoniae* as a key ARG carrier in PC patients, we designed culture conditions specifically to isolate and validate this species from tumor and pancreatic fluid samples. The successful recovery of 14 strains with concordant genomic features supports the targeted nature of our cultivation strategy.” Core genome-based phylogeny revealed close relatedness between PANC and PANF strains, indicating intra-patient spread. These isolates harbored the same resistance genes identified in metagenomic analysis, including efflux pump (*acrB*, *mdtC*) cells, regulators (*H-NS*, *cpxA*) (Nikaido, 2009; Paczosa and Mecsas, 2016), and membrane transport genes (*emr* family). These findings align with the concept of gut microbes seeding the tumor microenvironment via anatomical continuity or barrier disruption (Pushalkar et al., 2018; Riquelme et al., 2019). Tumor-associated hypervirulence loci (*rmpA*, *iucA*, *ybtA*) further suggest these bacteria may evade host defenses and persist in hostile environments (Lam et al., 2019). Consequently, our findings provided a focused validation of a key pathobiont but do not represent a comprehensive census of the viable tumor microbiota. Future studies employing multi-media culturomics and anaerobic techniques will be crucial to fully elucidate the ecological complexity and functional roles of the entire microbial community in PC. As the antibiotic treatment also required the involvement of adaptive immunity thereby improving the tumor microenvironment, suggesting that the action was not simply through a direct inhibitory effect on tumorigenesis (Horvat et al., 2024) Therefore, with consideration of ARGs, the presence of resistant *K. pneumoniae* in pancreatic fluid increases the risk of post-operative infections and may necessitate broader-spectrum prophylaxis.

While computational biology has successfully identified genetic biomarkers and immunomodulatory targets in PC (Kaviyaprabha et al., 2025; Tian et al., 2025), our findings have important implications for understanding cancer metastasis, chemoresistance management, and microbial biomarker development in PC. Through non-invasive fecal metagenomic screening, we identified PC patients carrying tumor-resident *K. pneumoniae* with coexisting ARGs and virulence factors, raising concerns about both postoperative infections and reduced chemotherapy efficacy. Our phylogenetic data support intra-patient spread and point to the need for gut microbiota surveillance. More importantly, by employing an innovative approach to track ARG transmission patterns, we provide compelling evidence that the translocation and intratumoral colonization of MDR *K. pneumoniae* implicates the gut-tumor axis as a clinically relevant pathway influencing both chemoresistance and immune microenvironment remodeling. This evidence supported a multidimensional therapeutic strategy integrating conventional treatments with microbiota-targeting interventions to overcome chemoresistance rooted in bacterial colonization. Future multi-center studies that also account for genetic influences will be essential to validate and generalize these findings. In conclusion, *K. pneumoniae* may act as a gut-derived pathobiont in PC, bridging microbial dysbiosis and therapeutic resistance.

## 5 Conclusion

By integrating metagenomic data with genomic evidence from isolates, this work provides direct evidence of the gut-to-tumor translocation of MDR K. pneumoniae in pancreatic cancer. The presence of these ARG-carrying strains links the gut microbiome to chemoresistance and postoperative infection risk, suggesting that fecal metagenomic monitoring could serve as a predictive tool for personalizing patient management.

## Data Availability

The datasets presented in this study can be found in online repositories. The names of the repository/repositories and accession number(s) can be found below: https://www.ncbi.nlm.nih.gov/, PRJNA1032163.
