# Supplementary material for: Gut-to-tumor translocation of multidrug-resistant Klebsiella pneumoniae shapes the microbiome and chemoresistance in pancreatic cancer
Source: Front Cell Infect Microbiol. 2025 Dec 2;15:1694479. doi: 10.3389/fcimb.2025.1694479 (PMC12705542; doi:10.3389/fcimb.2025.1694479)
Supplement: Supplementary file 1 [file Table1.docx]

Supplementary Material

# **Supplementary Tables**

**TABLE S1.** Raw data quality of Illumina sequencing.

| Sample ID | Clean Read | Clean Base | Clean Q20 * | Clean Q30 ** | Clean Date (GB) |
| --- | --- | --- | --- | --- | --- |
| A1 | 76619502 | 11470085904 | 97.53% | 92.97% | 11.47 |
| A2 | 81014434 | 12099292466 | 97.58% | 93.31% | 12.10 |
| A3 | 85789126 | 12813735662 | 97.78% | 93.61% | 12.81 |
| A4 | 83373956 | 12468007522 | 97.63% | 93.30% | 12.47 |
| A5 | 77621304 | 11619625320 | 96.61% | 91.24% | 11.62 |
| B1 | 67793612 | 10089177453 | 97.58% | 93.22% | 10.09 |
| B2 | 77038390 | 11514982476 | 97.78% | 93.66% | 11.51 |
| B3 | 70741350 | 10558797051 | 97.59% | 93.39% | 10.56 |
| B4 | 69646932 | 10429393882 | 97.62% | 93.16% | 10.43 |
| B5 | 73484784 | 10981340899 | 97.59% | 93.19% | 10.98 |
| CA1 | 70035824 | 10483041280 | 97.57% | 93.03% | 10.48 |
| CA2 | 101085168 | 15112077296 | 97.25% | 92.61% | 15.11 |
| CA3 | 74212572 | 11102694151 | 97.88% | 93.82% | 11.10 |
| CA4 | 74748684 | 11163177664 | 97.56% | 93.28% | 11.16 |
| CA5 | 69617640 | 10389594033 | 97.64% | 93.44% | 10.39 |
| CB1 | 77541590 | 11586143506 | 97.32% | 92.69% | 11.59 |
| CB2 | 84987888 | 12699107046 | 97.81% | 93.70% | 12.70 |
| CB3 | 77533416 | 11604521611 | 96.98% | 92.02% | 11.60 |
| CB4 | 69990492 | 10480494880 | 97.62% | 93.20% | 10.48 |
| CB5 | 86149364 | 12894029638 | 97.14% | 92.25% | 12.89 |

Note, * and ** representing the percentage of clean bases with a mass value of over 20% and 30%, respectively.

**TABLE S2.** The contigs of assembled reads from all the samples (more than 500bp).

| Sample ID | Total number | Total length* | Avg. length | Max. length | N50 length | GC (%) |
| --- | --- | --- | --- | --- | --- | --- |
| A1 | 49669 | 173123394 | 3485.54 | 1243804 | 23048 | 47.82 |
| A2 | 100004 | 236133841 | 2361.24 | 538790 | 5978 | 47.01 |
| A3 | 81742 | 224479373 | 2746.19 | 813141 | 13101 | 47.67 |
| A4 | 120743 | 286308051 | 2371.22 | 797702 | 6672 | 48.96 |
| A5 | 80320 | 200688203 | 2498.61 | 610552 | 8122 | 47.28 |
| B1 | 67893 | 173571965 | 2556.55 | 575347 | 10516 | 47.77 |
| B2 | 29817 | 97270982 | 3262.27 | 497901 | 13452 | 44.9 |
| B3 | 53205 | 158763439 | 2983.99 | 746212 | 14163 | 48.38 |
| B4 | 58265 | 134430073 | 2307.22 | 587353 | 8892 | 47 |
| B5 | 154881 | 334233252 | 2158 | 702313 | 5548 | 46.78 |
| CA1 | 30003 | 99857274 | 3328.24 | 544425 | 32120 | 43.92 |
| CA2 | 63179 | 135633935 | 2146.82 | 727136 | 4989 | 45.1 |
| CA3 | 71214 | 120771719 | 1695.9 | 333756 | 2793 | 46.78 |
| CA4 | 137598 | 294601757 | 2141.03 | 396785 | 4829 | 48.47 |
| CA5 | 136028 | 325139731 | 2390.24 | 626671 | 6606 | 48.33 |
| CB1 | 48634 | 148262322 | 3048.53 | 606468 | 14522 | 45.4 |
| CB2 | 122034 | 301527486 | 2470.85 | 777902 | 10214 | 47.94 |
| CB3 | 49479 | 175637016 | 3549.73 | 695085 | 32300 | 46.19 |
| CB4 | 82817 | 155956946 | 1883.15 | 359750 | 3401 | 47.19 |
| CB5 | 23678 | 98188897 | 4146.84 | 387385 | 32741 | 44.04 |

Note, * the unit of length in the table is bp.

**TABLE S3.** The significant correlations between the targeted bacterial species and ARGs conducted in fecal samples of MPC group.

| Variable1 | Variable2 | PearsonR | PearsonPvalue | adjPearsonP |
| --- | --- | --- | --- | --- |
| *Akkermansia_muciniphila* | tet32 | 0.950225 | 0.013231 | 0.023738 |
| *Akkermansia_muciniphila* | tet_40 | 0.904631 | 0.034845 | 0.036182 |
| *Akkermansia_muciniphila* | lnuC | 0.989324 | 0.001322 | 0.01074 |
| *Akkermansia_muciniphila* | ErmB | 0.916572 | 0.028562 | 0.032925 |
| *Akkermansia_muciniphila* | CfxA3 | 0.91404 | 0.029858 | 0.033695 |
| *Akkermansia_muciniphila* | tetO | 0.949748 | 0.01342 | 0.023854 |
| *Akkermansia_muciniphila* | APH_2''_If | 0.97655 | 0.004295 | 0.015858 |
| *Klebsiella_oxytoca* | acrB | 0.919177 | 0.027246 | 0.032267 |
| *Klebsiella_oxytoca* | adeF | 0.90383 | 0.035278 | 0.036445 |
| *Klebsiella_oxytoca* | baeR | 0.95755 | 0.010432 | 0.021654 |
| *Klebsiella_oxytoca* | mdtC | 0.938609 | 0.018091 | 0.027017 |
| *Klebsiella_pneumoniae* | PmrF | 0.974045 | 0.005 | 0.016641 |
| *Klebsiella_pneumoniae* | Resistance_to_Pulvomycin | 0.978139 | 0.003867 | 0.015114 |
| *Klebsiella_pneumoniae* | mdtC | 0.941678 | 0.016759 | 0.026208 |
| *Klebsiella_pneumoniae* | H_NS | 0.932007 | 0.021065 | 0.028853 |
| *Klebsiella_pneumoniae* | acrB | 0.95163 | 0.012677 | 0.023321 |
| *Klebsiella_pneumoniae* | cpxA | 0.972485 | 0.005456 | 0.017241 |

**TABLE S4.** The significant correlations between the targeted bacterial species and ARGs conducted in fecal samples of NMPC.

| Variable1 | Variable2 | PearsonR | PearsonPvalue | adjPearsonP |
| --- | --- | --- | --- | --- |
| s_Klebsiella_pneumoniae | tet_W_N_W_ | 0.96859 | 0.00665 | 0.014433 |
| s_Klebsiella_pneumoniae | mdtF | 0.997751 | 0.000128 | 0.003812 |
| s_Klebsiella_pneumoniae | emrK | 0.995729 | 0.000335 | 0.004751 |
| s_Klebsiella_pneumoniae | ErmF | 0.95768 | 0.010384 | 0.018282 |
| s_Klebsiella_pneumoniae | msbA | 0.986385 | 0.001903 | 0.008457 |

**TABLE S5.** The results of identified ARGs checked against NT and Refseq database

****Abbrevia**tions:** ARGs (antibiotic resistance genes). ARGs were identified by aligning metagenomic reads against the CARD database, followed by taxonomic assignment of ARG-carrying contigs using Kraken2. After refining the Host-ARG associations, the identified ARGs were cross-checked against two additional databases: NT(Nucleotide Sequence Database) and RefSeq (NCBI Reference Sequence Database). The bolded gene names represent the ARGs that were confirmed in these databases.

## **Supplementary Figures**


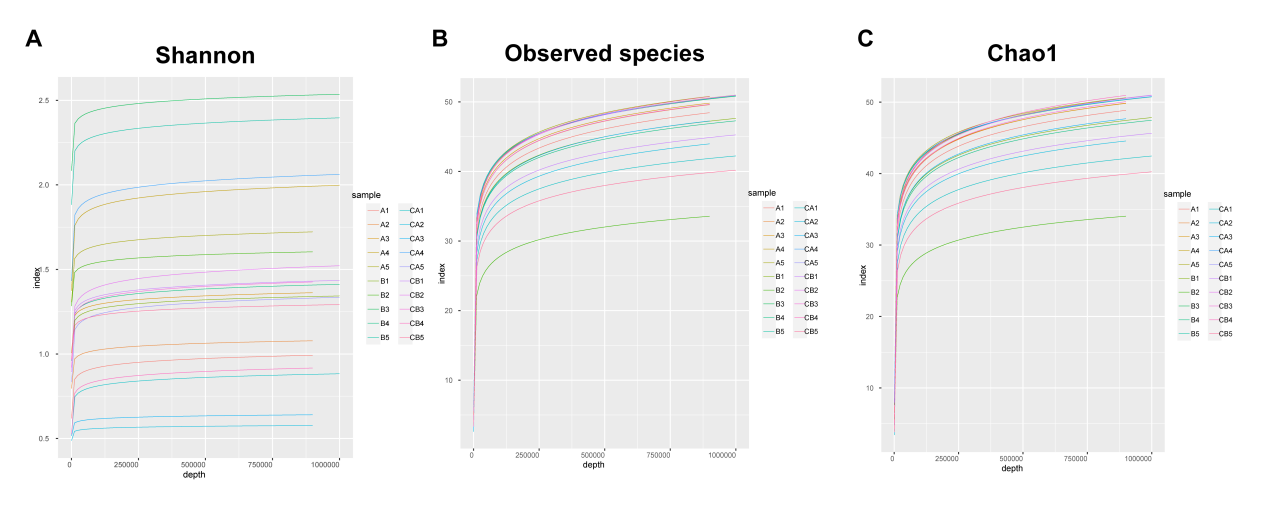


**Supplementary Figure 1.** Rarefaction curves based on the (A) Shannon index, (B) observed species, and (C) Chao1 estimator.


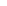


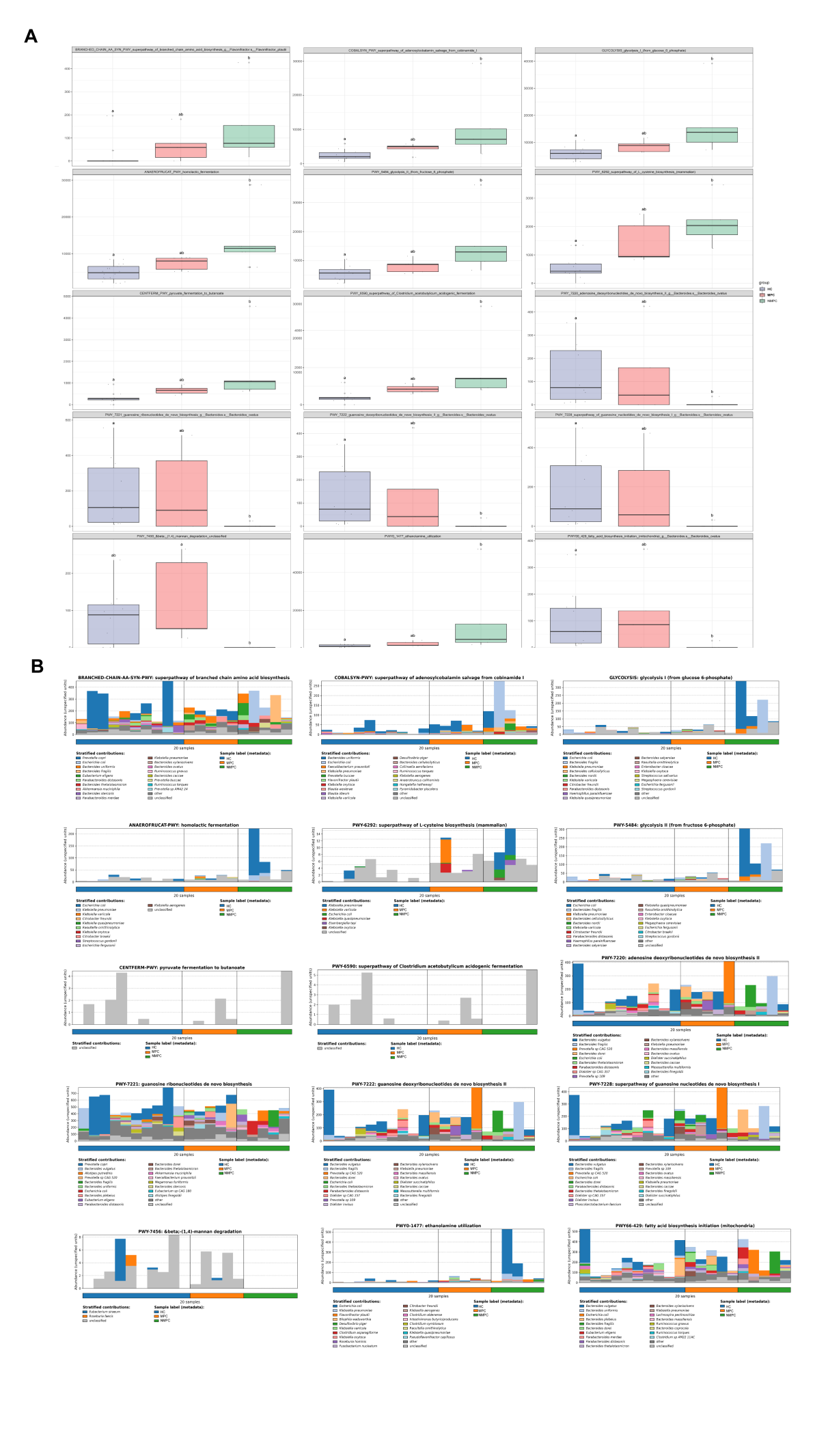


**Supplementary Figure 2.** Relative abundance of other15 differently expressed metabolic pathways and their stratified contributions. (A) 15 differently expressed metabolic pathways; (B) corresponding stratified contributions.There was a significant difference between a and b in each pathway.
